# PRPS2‐mediated modulation of the antitumor immune response in lung cancer through CCL2‐mediated tumor‐associated macrophages and myeloid‐derived suppressor cells

**DOI:** 10.1111/1759-7714.15398

**Published:** 2024-07-01

**Authors:** Qing Liu, Ningzi Wu, Peifeng Hou

**Affiliations:** ^1^ Department of Oncology Fujian Medical University Union Hospital Fuzhou China; ^2^ Fujian Key Laboratory of Translational Cancer Medicine Fuzhou China; ^3^ Fujian Medical University Fuzhou China

**Keywords:** CCL2, lung cancer, macrophages, myeloid‐derived suppressor cells, PRPS2

## Abstract

**Background:**

Phosphoribosyl pyrophosphate synthetase 2 (PRPS2) is known as an oncogene in many types of cancers, including lung cancer. However, its role in regulating tumor‐associated macrophages (TAM) and myeloid‐derived suppressor cells (MDSC) remains unclear. Our study aimed to explore the involvement of PRPS2 in TAM and MDSC regulation.

**Methods:**

Stable Lewis lung cancer (LLC) cell lines were established using a lentivirus system. These LLC lines were then used to establish tumor model in mice. The levels of target genes were determined using qPCR, western blotting, and ELISA assays. The percentage of different immune cell types was analyzed using fluorescence‐activated cell sorting. The chemotaxis ability of TAM and MDSC was evaluated using an in vitro transwell chemotaxis assay.

**Results:**

Notably, PRPS2 was found to regulate the chemotaxis of TAM and MDSC in tumor cells, as evidenced by the positive correlation of PRPS2 expression levels and abundance of TAM and MDSC populations. In addition, the expression of CCL2, mediated by PRPS2, was identified as a key factor in the chemotaxis of TAM and MDSC, as evidenced by a significant reduction in macrophages and MDSC numbers in the presence of the CCL2 antibody. Furthermore, in vivo experiments confirmed the involvement of PRPS2 in mediating CCL2 expression. PRPS2 was also found to regulate immune cell infiltration into tumors, whereas knockdown of CCL2 reversed the phenotype induced by PRPS2 overexpression. In tumor tissues from mice implanted with LLC‐PRPS2‐shCCL2 cells, a notable increase in CD4+ and CD8+ T cell percentages, alongside a marked decrease in TAMs, M‐MDSC, and PMN‐MDSC, was observed.

**Conclusion:**

Taken together, PRPS2 plays a crucial role in modulating the antitumor immune response by reprogramming CCL2‐mediated TAM and MDSC.

## Graphical abstract

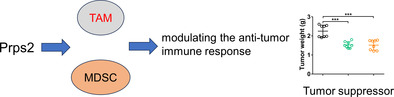

## INTRODUCTION

Lung cancer is a pervasive malignancy with a global impact.^1^ Recent estimates from GLOBOCAN in 2018 revealed an incidence of approximately 2 094 000 new cases worldwide.^2^ Lung cancer is the second most prevalent cancer in both men (1 369 000 cases) and women (725 000 cases).^2^ The five‐year relative survival rate for all subtypes of lung cancer, including non‐small cell lung cancer and small cell lung cancer, is a mere 19%.^3^ These statistics highlight the pressing need to explore novel therapeutic strategies for lung cancer, aiming to significantly improve patient outcomes and survival rates.

In addition to cancer cells, the tumor microenvironment consists of a diverse array of immune cells, including T cells, tumor‐associated macrophages (TAM), and myeloid‐derived suppressor cells (MDSC).^4^, ^5^, ^6^ The intricate interplay between cancer cells and immune cells is crucial for shaping the tumor immune landscape, exerting significant effects on antitumor immune responses, tumor cell proliferation, and metastasis.^5^, ^7^ TAM, as a predominant immune cell subset infiltrating tumors, exhibit remarkable phenotypic plasticity and can be broadly categorized into two distinctive polarization named M1 and M2 macrophages.^7^, ^8^ M1 macrophages display potent antitumor effector functions, including cytotoxicity and antibody‐dependent cell‐mediated cytotoxicity (ADCC), which contribute to the elimination of malignant cells. Conversely, M2 macrophages promote tumor growth, metastatic dissemination, angiogenesis, and dampen T cell‐mediated immune responses.^7^ Additionally, within the tumor microenvironment, various populations of polymorphonuclear cells (PMN), including classical PMN, polymorphonuclear myeloid‐derived suppressor cells (PMN‐MDSC), and activated PMN‐MDSC, contribute to the immunosuppressive activities observed in tumor tissues.^9^, ^10^ Therefore, targeting the chemotaxis of TAM and MDSC represents a promising therapeutic strategy for antitumor therapy.

Phosphoribosyl pyrophosphate synthetase 2 (PRPS2) has emerged as an oncogenic factor in various cancer types, supported by its documented involvement in promoting carcinogenesis.^11^ Previous in vitro studies from our group have unveiled the role of PRPS2 in enhancing cisplatin resistance in lung cancer through the promotion of M2 macrophage polarization.^12^ Nevertheless, the regulatory impact of PRPS2 on TAM and MDSC within the in vivo context, particularly in immunocompetent mice, remains largely unexplored. Notably, TAM and MDSC hold critical significance within the tumor microenvironment by exerting potent immune suppressive effects and facilitating tumor progression. Intriguingly, CCL2, a potent chemokine known to attract monocytes and various immune cell populations, has been identified as a pivotal chemotactic factor involved in the recruitment of TAM and MDSC into tumor tissues.^13^ In detail, CCL2 is recognized for its role in recruiting TAMs to tumor tissues and influencing their polarization, as well as MDSC. More importantly, the CCR2/CCL2 axis is required for MDSC and TAM functional specialization.^13^ However, it remains unclear whether the regulatory roles of PRPS2 in TAMs and MDSC are linked to CCL2. Herein, we aimed to shed light on the roles of PRPS2 in regulating the recruitment of TAM and MDSC, while also investigating the potential interplay between PRPS2 and CCL2 in the context of lung cancer. Our results provided novel insights into the crucial role of PRPS2 in modulating the anticancer immune response through the reprogramming of CCL2‐mediated TAM and MDSC functions.

## METHODS

### Cell culture

Lewis lung cancer (LLC) cell lines were obtained from the ATCC and cultured in Dulbecco's modified Eagle medium (DMEM) supplemented with 10% fetal bovine serum (FBS, Hyclone) and 10 μg/mL penicillin–streptomycin (Gibco). Cells were cultured at 37°C in a humidified atmosphere with 5% CO_2_.

### Western blotting

Western blotting was conducted as previously described.^14^ The following primary antibodies were used including PRPS2 antibody (ThermoFisher, cat no: PA5‐42007; at a dilution of 1:2000), CCL2 antibody (Abcam, cat no: ab25124; at a dilution of 1:1000), and glyceraldehyde 3‐phosphate dehydrogenase (GAPDH) antibody (CST, cat no: 2118; at a dilution of 1:5000). Briefly, 50 μg of protein was subjected to electrophoresis on a 12% (v/v) SDS‐polyacrylamide gel. Subsequently, the protein was transferred onto a polyvinylidene fluoride (PVDF) membrane through electroblotting. To prevent nonspecific binding, the membrane was exposed to a solution of phosphate buffered saline (PBS) containing 5% non‐fat milk for 1 h at room temperature. Subsequently, the membrane was incubated with primary antibodies overnight at 4°C. Following the primary antibody incubation, a horseradish peroxidase‐conjugated secondary antibody was applied to the membrane for 1 h. After three washes, the membrane was visualized using an enhanced chemiluminescence system.

### Quantitative real‐time polymerase chain reaction (qRT‐PCR)

The qRT‐PCR method was performed as previously described.^14^ The primer sequences used are as follows: *PRPS2*: Forward: 5′‐ATG CCTAACATCGTGCTCTTC‐3′, Reverse: 5′‐GATCTCGACACTGGTCTCCTG‐3′; *CCL2*: Forward: 5’‐CAGCCAGATGCAATCAATGCC‐3′, Reverse: 5′‐TGGAATCCTGAACCCACTTCT‐3′; *S100A8*: Forward:5′‐AAATCACCATGCCCTCTACAAG‐3′, Reverse: 5′‐CCCACTTTTATCACCATCGCAA‐3′; *S100A9*: Forward: 5′‐ATACTCTAGGAAGGAAGGACACC‐3′, Reverse: 5′‐TCCATGATGTCATTTATGAGGGC‐3′; *Nos2*: Forward: 5′‐GGAGTGACGGCAAACATGACT‐3′, Reverse: 5′‐TCGATGCACAACTGGGTGAAC‐3′; *Arg1*: Forward: 5′‐CTCCAAGCCAAAGTCCTTAGAG‐3′, Reverse: 5′‐GGA GCTGTCATTAGGGACATCA‐3′; *GAPDH*: Forward: 5′‐AGGTCGGTGTGAACGGATTTG‐3′, Reverse: 5′‐TGTAGACCATGT AGTTGAGGTCA‐3′. In brief, TRIzol reagent was used to extract total RNA. To synthesize complementary DNA (cDNA), 1 μg of total RNA was added to the SuperScript master mix, and reverse transcription was conducted. Quantitative PCR was performed using SYBR Green Supermix (Bio‐Rad, USA). The expression levels of the genes of interest in different samples were determined using the comparative Ct value method. The mRNA levels were normalized to the reference gene *GAPDH*, which served as the internal control.

### Stable cell line construction

LLC stable cell lines were established through the utilization of lentiviral vectors.^15^ Specifically, PLKO.1‐TRC (Addgene) was employed to generate lentiviral vectors encoding shRNAs, namely shPRPS2 (Sigma, TRCN0000025543), LLC‐shCCL2 (Sigma, TRCN0000034470), and shControl (Sigma, SHC016). In addition, pLV‐puro (Addgene) was utilized to construct lentiviral vectors containing the mouse *PRPS2* gene. LLC cells transduced with the control virus served as the negative control and were designated as LLC. The production of lentivirus and subsequent cell infection were carried out following the recommended lentiviral vector protocol provided by Addgene. Infected cells were selected using puromycin for a period of 2 weeks to establish stable cell lines.

### In vitro transwell chemotaxis assay

The in vitro chemotaxis assay for TAM and MDSC was performed as referenced.^16^ TAM (F4/80^+^CD206^+^) and MDSC (CD11b^+^Gr‐1^+^) were isolated from LLC mouse tumor using flow cytometry. Then, 105 TAM or MDSC were added to the upper chambers of transwell plates with 5‐μm pore inserts (Corning). Supernatants from various LLC, LLC‐PRPS2, LLC‐shPRPS2 cells were added to the lower chambers with or without neutralizing anti‐CCL2 antibody (500 ng/mL, eBioscience). After a 4‐h incubation, the migrated cells in the lower chambers were counted using a hemocytometer.

### Fluorescence‐activated cell sorting analysis

The method for preparing single‐cell suspensions from tumor tissue and the fluorescence‐activated cell sorting (FACS) experiment were performed as previously described.^15^ All of the antibodies for the FACS experiment were purchased from Biolegend. Specific details regarding the antibodies used can be found in the reference.

### ELISA

The Ccl2 enzyme‐linked immunosorbent assay (ELISA) method was performed as previously reported.^14^ In brief, to ensure uniform protein quantities, the protein content was determined using a bicinchoninic acid (BCA) assay (Thermo Scientific). CCL2 levels were then assessed using an ELISA assay following the instructions provided by the manufacturer (R&D Systems).

### LLC tumor model construction and drug treatment

The LLC tumor model and drug treatment methods were performed as previously reported.^17^ The different stable cell lines were cultured and subsequently implanted subcutaneously into C57BL/6 mice (female, 6–8 week‐old). Tumor growth was measured using calipers on the dimensions, and the tumor volume was calculated according to these measurements. To deplete macrophages, clodronate liposomes (200 μL, FormuMax Scientific Inc., USA) or empty liposomes were injected intraperitoneally into the mice 24 h prior to the implantation of tumor cells, and subsequently every 5 days. For depletion of MDSC, anti‐Gr‐1 antibody (2 mg/kg, twice weekly) was administered via intraperitoneal injection. Animal studies were approved by Fujian Medical University Union Hospital.

### Statistical analysis

One‐way analysis of variance (ANOVA) or two‐way ANOVA tests were conducted in GraphPad Prism 8. The error bars represent the mean ± standard deviation (SD). A statistical significance was concluded as follows: *p < 0.05, **p < 0.01, ***p < 0.001, ns, not significant.

## RESULTS

### PRPS2 regulated chemotaxis of TAM and MDSC in tumor cells

**FIGURE 1:**
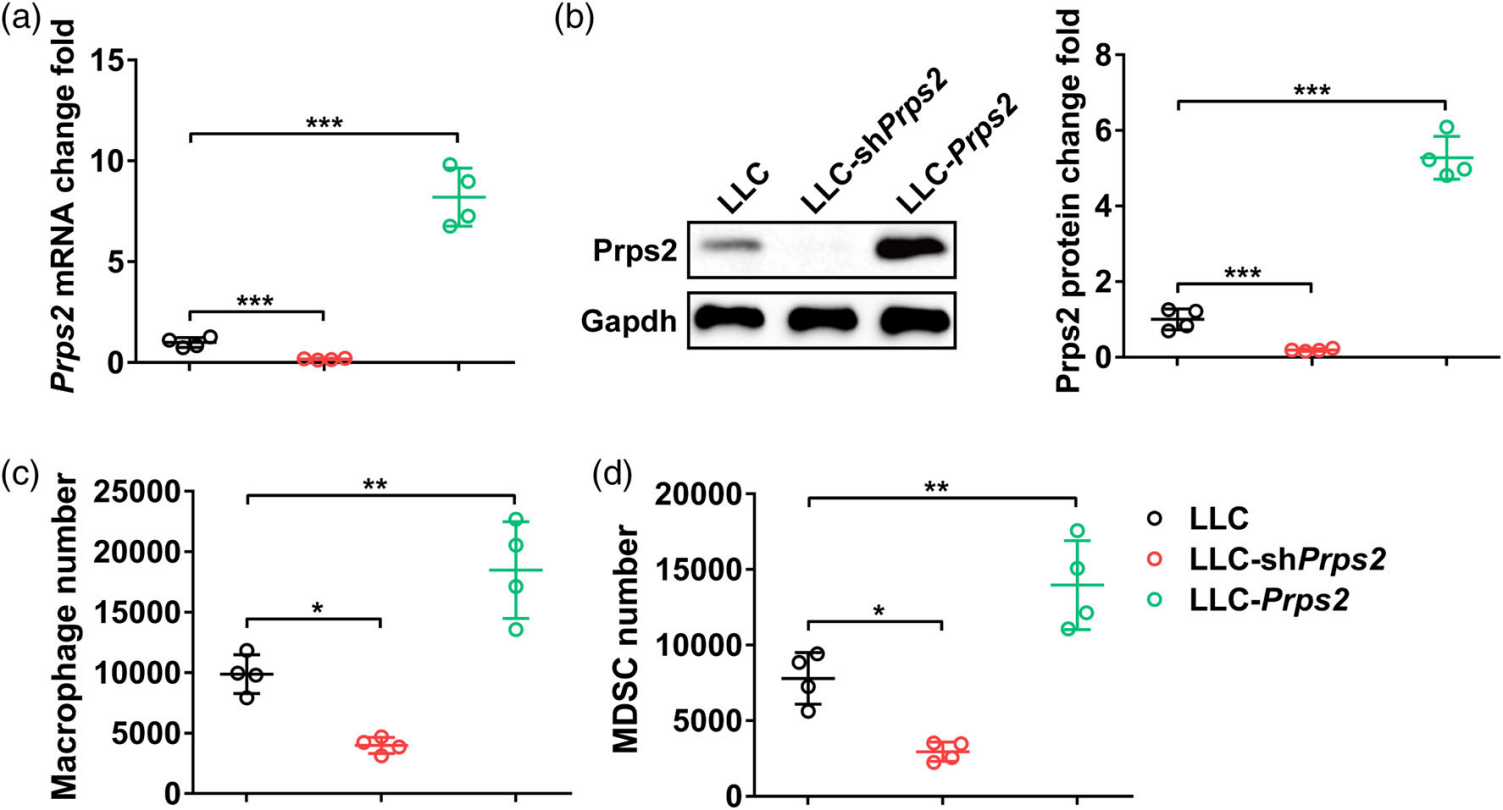
PRPS2 regulates chemotaxis of tumor‐associated macrophages (TAM) and myeloid‐derived suppressor cells (MDSC) in tumor cells. (a) Relative mRNA and (b) protein levels of PRPS2 in control Lewis lung cancer (LLC), LLC‐shPRPS2, and LLC‐PRPS2 cells. (c) Number of migrated macrophages and (d) MDSC in a transwell chemotactic assay. Four wells per group. Statistical significance was analyzed using one‐way analysis of variance (ANOVA).

In this study, we successfully manipulated the expression of PRPS2 in tumor cells, as evidenced by the corresponding decrease/increase in PRPS2 mRNA levels (Figure 1a) and protein levels (Figure 1b) in LLC cells transfected with shPRPS2 or PRPS2‐containing plasmid, respectively. Interestingly, we made an intriguing observation that the migration of macrophages (Figure 1c) was significantly reduced, while the number of MDSC cells (Figure 1d) was elevated. These findings indicated that PRPS2 plays a regulatory role in the chemotaxis of TAM and MDSC within the tumor microenvironment.

### PRPS2‐mediated CCL2 in tumor cells regulates chemotaxis of TAM and MDSC

**FIGURE 2:**
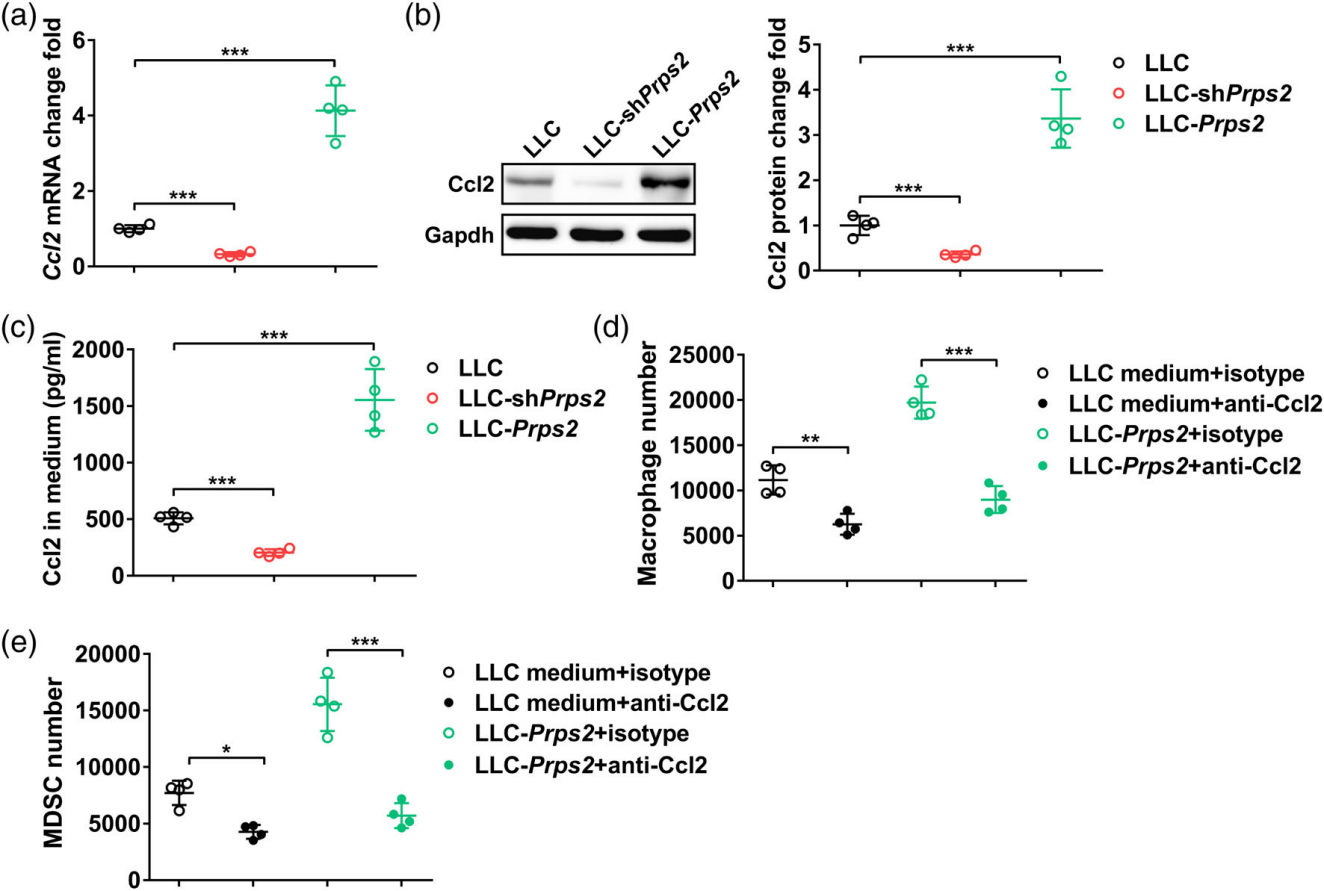
PRPS2‐mediated CCL2 in tumor cells regulates chemotaxis of tumor‐associated macrophages (TAM) and myeloid‐derived suppressor cells (MDSC). (a) Relative mRNA and (b) protein levels of Ccl2 in control Lewis lung cancer (LLC), LLC‐shPRPS2, and LLC‐PRPS2 cells. (c) CCL2 protein level in the culture medium of control LLC, LLC‐shPRPS2, and LLC‐PRPS2 cells. (d) Number of migrated macrophages and (e) MDSC in a transwell chemotactic assay using isotype or anti‐CCL2 antibody. Four wells per group. Statistical significance was analyzed using one‐way analysis of variance (ANOVA).

In order to elucidate the underlying mechanism by which PRPS2 regulates the chemotaxis of TAM and MDSC, we conducted further investigations to explore the relationship between PRPS2 and the chemokine CCL2. A positive correlation between PRPS2 and CCL2 was observed, as suggested by the decrease in CCL2 levels at the mRNA (Figure 2a), intracellular (Figure 2b), and extracellular levels (Figure 2c) upon PRPS2 knockout, while PRPS2 overexpression led to an increase in CCL2 levels (Figure 2a–c). To validate the association between PRPS2 and CCL2, we introduced a CCL2 antibody into the system. Interestingly, we observed a significant reduction in the number of macrophages (Figure 2d) and MDSC (Figure 2e) in the presence of the CCL2 antibody, suggesting that PRPS2‐mediated regulation of CCL2 in tumor cells plays a crucial role in the chemotaxis of TAMs and MDSC.

### PRPS2 mediates CCL2 expression

**FIGURE 3:**
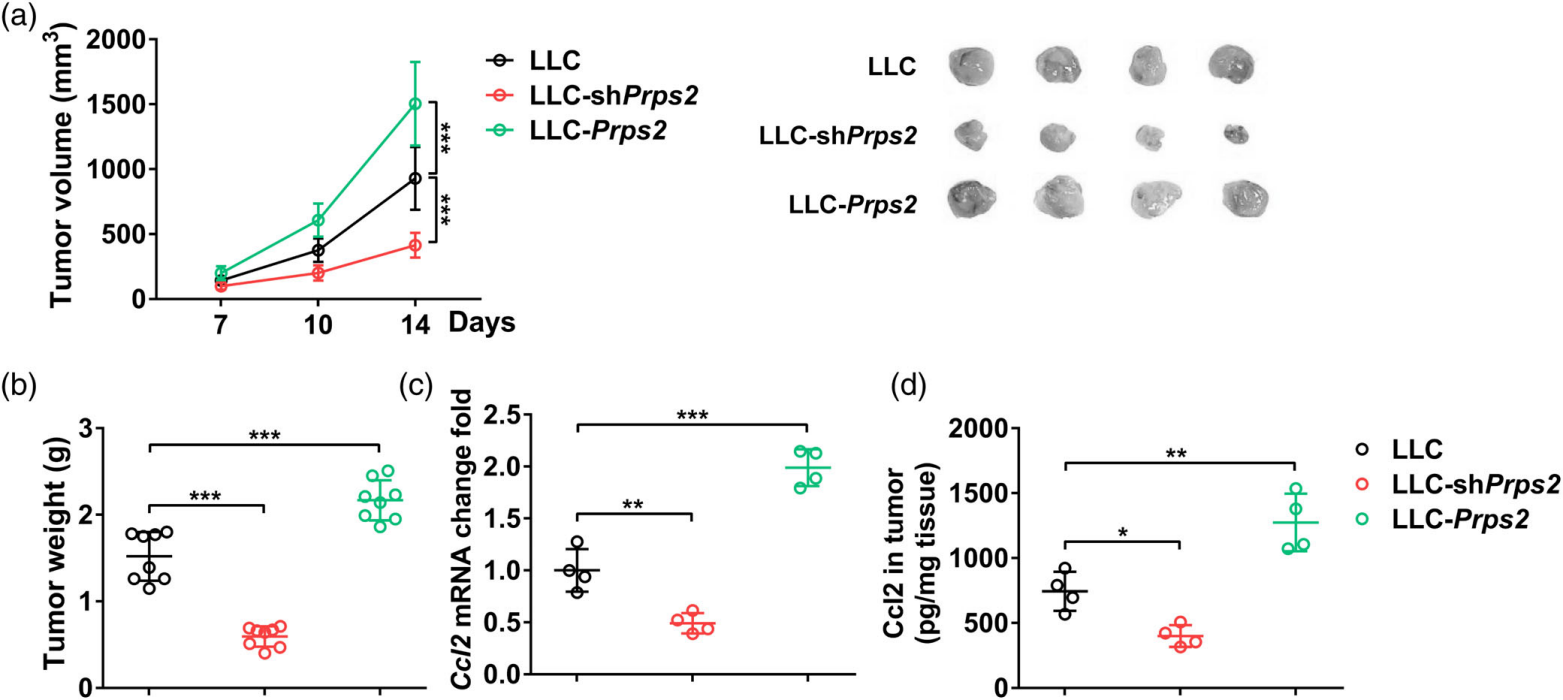
PRPS2 mediates CCL2 expression in vivo. (a) Lewis lung cancer (LLC) tumor volume at day 7, 10, and 14, and representative tumor images at day 14. Statistical significance was analyzed using two‐way analysis of variance (ANOVA). (b) Tumor weight at day 14. Eight mice per group. (c) The mRNA and (d) protein levels of CCL2 in control LLC, LLC‐shPRPS2, and LLC‐PRPS2 tumor tissues. Four mice per group. Statistical significance was analyzed using one‐way ANOVA.

We conducted an in vivo animal study to investigate the relationship between PRPS2 and CCL2. First, we observed a significant decrease in both tumor volume and tumor weight in mice implanted withLLC‐shPRPS2 cells (Figure 3a). Conversely, mice implanted withLLC‐PRPS2 cells displayed an increase in tumor volume and tumor weight (Figure 3b). Intriguingly, we also observed a similar trend in CCL2 levels, both at the mRNA and protein levels, in tumor tissues (Figure 3c and d), further confirming the positive correlation between PRPS2 and CCL2 in the LLC tumor tissues.

### PRPS2 regulates immune cell infiltration into tumors

**FIGURE 4:**
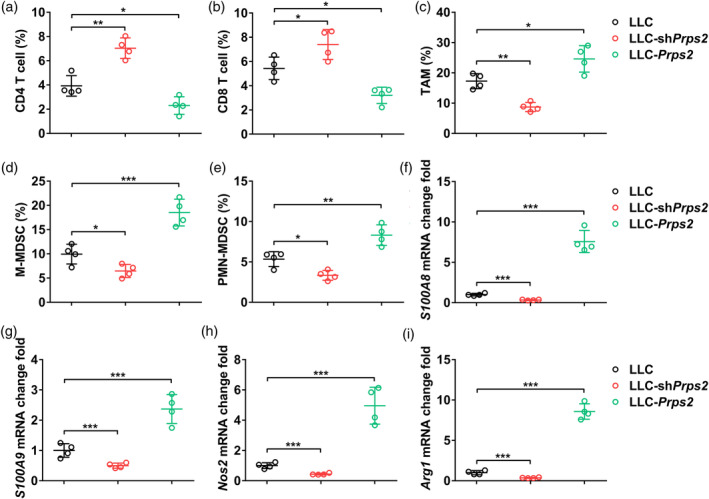
PRPS2 regulates immune cell infiltration into tumors. Percentage of (a) CD4 T cells (CD3^+^CD4^+^), (b) CD8 T cells (CD3^+^CD8^+^), (c) tumor‐associated macrophages (TAM) (CD45^+^F4/80^+^CD206^+^), (d) M‐MDSC (CD45^+^CD11b^+^Ly6C^+^), and (e) PMN‐MDSC (CD45^+^CD11b^+^Ly6G^+^) in control Lewis lung cancer (LLC), LLC‐shPRPS2, and LLC‐PRPS2 tumor tissues at day 14. Relative mRNA levels of (f) S100A8, (g) S100A9, (h) Nos2, and (i) Arg1 in control LLC, LLC‐shPRPS2, and LLC‐PRPS2 tumor tissues at day 14. Four mice per group. Statistical significance was analyzed using one‐way analysis of variance (ANOVA).

We then proceeded to investigate the impact of PRPS2 on immune cell infiltration into tumors by examining the percentage of T cells (CD4+ and CD8+), TAM, and MDSC, as well as their associated biomarkers. Remarkably, in the tumor tissues of mice implanted with LLC‐shPRPS2 cells, we observed a significant increase in the percentage of CD4+ T cells (Figure 4a) and CD8+ T cells (Figure 4b), along with a notable reduction in the percentage of TAM (Figure 4c), M‐MDSC (Figure 4d), and PMN‐MDSC (Figure 4e). Conversely, in the tumor tissues derived from mice implanted with LLC‐PRPS2 cells, we observed a significant decrease in the percentage of CD4+ T cells (Figure 4a) and CD8+ T cells (Figure 4b), coupled with a significant increase in the percentage of TAMs (Figure 4c), M‐MDSC (Figure 4d), and PMN‐MDSC (Figure 4e).

Furthermore, we assessed the relative mRNA levels of S100A8 (Figure 4f), S100A9 (Figure 4g), Nos2 (Figure 4h), and Arg1 (Figure 4i) in the tumor tissues. Notably, we found that the mRNA levels of these biomarkers were significantly decreased in the tumor tissues of mice implanted with LLC‐shPRPS2 cells, whereas they were significantly increased in the tumor tissues of mice implanted with LLC‐PRPS2 cells. Collectively, these findings provide evidence that PRPS2 may play a role in regulating immune cell infiltration into tumors.

### Knockdown of CCL2 reversed the phenotype of PRPS2 overexpression

**FIGURE 5:**
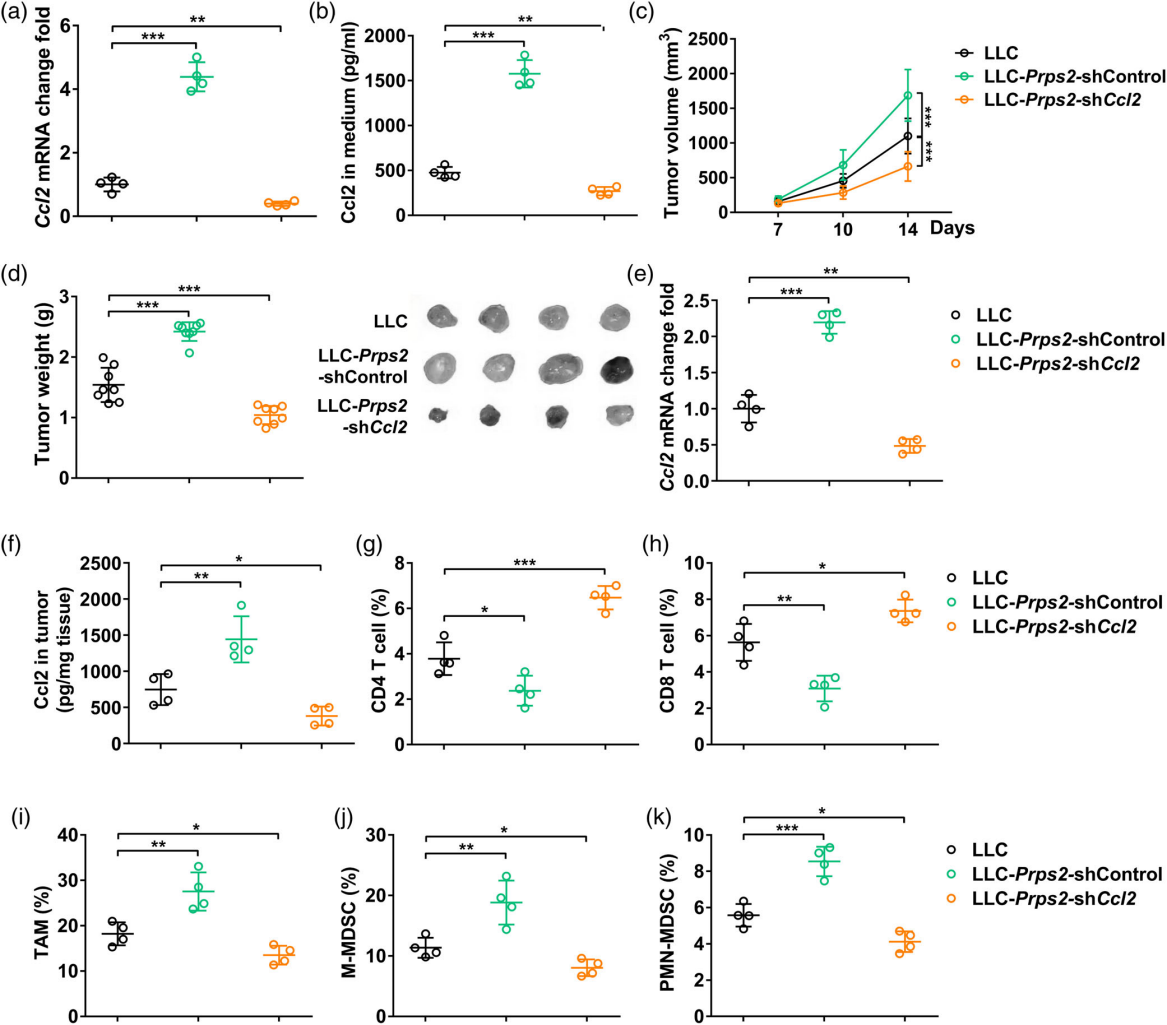
Knockdown of CCL2 reverses the phenotype of PRPS2 overexpression. (a) Relative mRNA level of CCL2 in control Lewis lung cancer (LLC), LLC‐PRPS2‐shControl, and LLC‐PRPS2‐shCCL2 cells in vitro. (b) CCL2 protein level in the culture medium of control LLC, LLC‐PRPS2‐shControl, and LLC‐PRPS2‐shCCL2 cells. Four wells per group. Statistical significance was analyzed using one‐way analysis of variance (ANOVA). (c) LLC tumor volume at day 7, 10, and 14. Statistical significance was analyzed using two‐way ANOVA. (d) Tumor weight and representative tumor images at day 14. Eight mice per group. The (e) mRNA and (f) protein levels of Ccl2 in control LLC, LLC‐PRPS2‐shControl, and LLC‐PRPS2‐shCCL2 tumor tissues. (g) Percentage of CD4 T cells (CD3^+^CD4^+^), (h) CD8 T cells (CD3^+^CD8^+^), (i) tumor‐associated macrophages (TAM) (CD45^+^F4/80^+^CD206^+^), (j) M‐MDSC (CD45^+^CD11b^+^Ly6C^+^) and (k) PMN‐MDSC (CD45^+^CD11b^+^Ly6G^+^) in control LLC, LLC‐PRPS2‐shControl, and LLC‐PRPS2‐shCcl2 tumor tissues at day 14. Four mice per group. Statistical significance was analyzed using one‐way ANOVA.

To further validate the positive correlation between PRPS2 and CCL2, we conducted in vitro experiments by xenografting mice with LLC‐PRPS2‐shCCL2 cells. Initially, we successfully achieved CCL2 knockdown, as evidenced by a significant decrease in CCL2 mRNA levels (Figure 5a) and secretion (Figure 5b) in the LLC‐PRPS2‐shCCL2 cells. Subsequently, we observed a notable reduction in both tumor volume and tumor weight in mice implanted with LLC‐PRPS2‐shCCL2 cells (Figure 5c and d). As expected, we also observed a similar trend in CCL2 levels, both at the mRNA and protein levels, in tumor tissues (Figure 5e,f). The mRNA and protein expression levels of Ccl2 were both significantly reduced in the LLC‐PRPS2‐shCCL2 cells. Additionally, in the tumor tissues of mice implanted with LLC‐PRPS2‐shCCL2 cells, we observed a significant increase in the percentage of CD4+ T cells (Figure 5g) and CD8+ T cells (Figure 5h), accompanied by a significant decrease in the percentage of TAMs (Figure 5i), M‐MDSC (Figure 5j), and PMN‐MDSC (Figure 5k). These findings suggested that knockdown of Ccl2 reversed the phenotype associated with PRPS2 overexpression.

### Depletion of TAM and MDSC reverses the phenotype of PRPS2 overexpression

**FIGURE 6:**
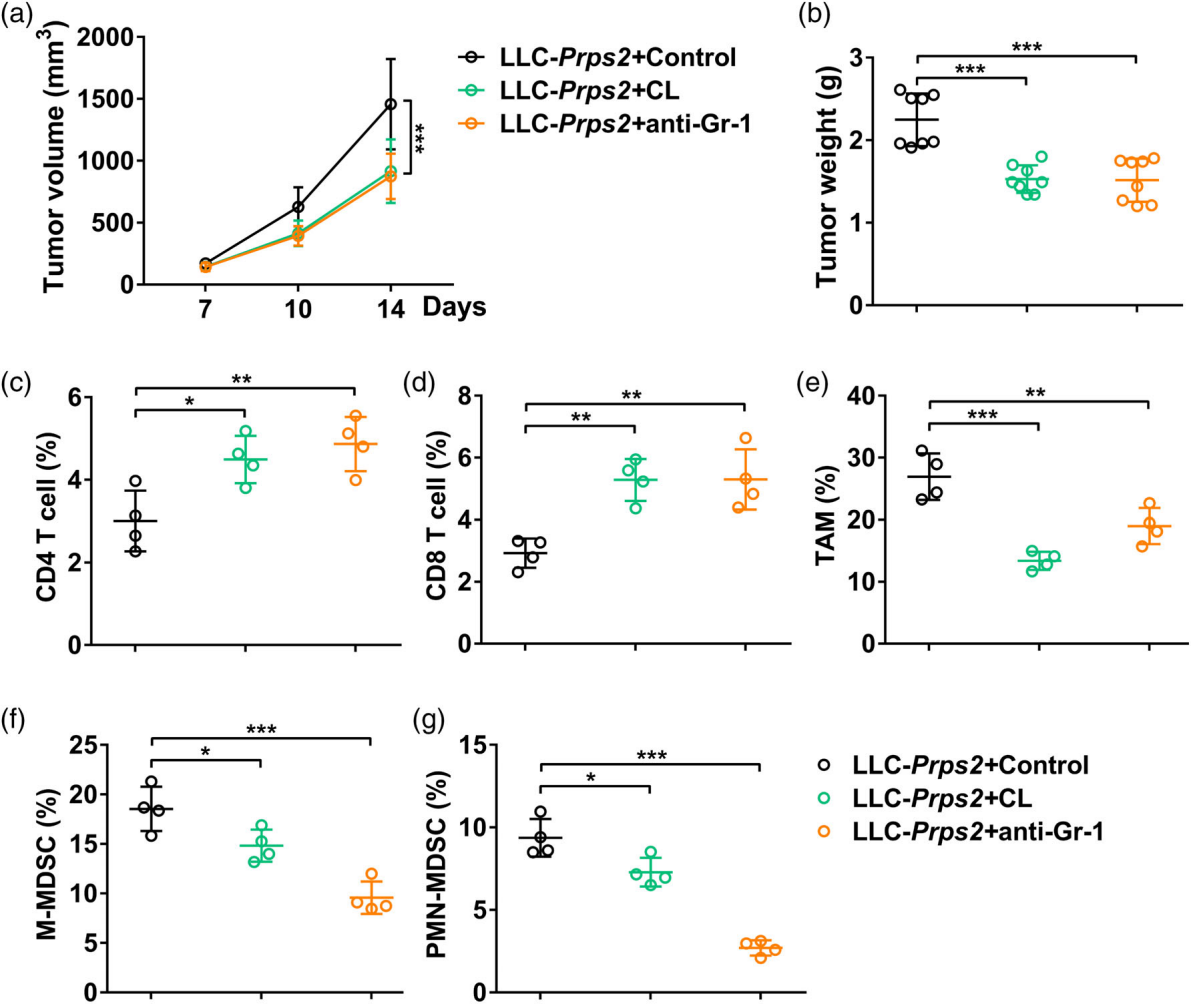
Depletion of tumor‐associated macrophages (TAM) and myeloid‐derived suppressor cells (MDSC) reverses the phenotype of PRPS2 overexpression. (a) Lewis lung cancer (LLC) tumor volume at day 7, 10, and 14. (b) Tumor weight at day 14. Eight mice per group. (c) Percentage of CD4 T cells (CD3^+^CD4^+^), (d) CD8 T cells (CD3^+^CD8^+^), (e) TAM (CD45^+^F4/80^+^CD206^+^), (f) M‐MDSC (CD45^+^CD11b^+^Ly6C^+^) and (g) PMN‐MDSC (CD45^+^CD11b^+^Ly6G^+^) in LLC‐PRPS2 tumor tissues at day 14 from mice treated with control reagent (control liposomes and isotype antibody), clodronate liposomes (CL), or anti‐Gr‐1 antibody. Four mice per group. Statistical significance was analyzed using one‐way analysis of variance (ANOVA).

To investigate the impact of depleting TAMs and MDSC on the phenotype resulting from PRPS2 overexpression, we utilized clodronate liposomes (CL) and anti‐Gr‐1 antibody in a xenograft animal model. Remarkably, the TAM and MDSC depletion groups exhibited a significant reduction in both tumor volume and tumor weight (Figure 6a,b).

We then assessed the composition of immune cells within the tumor microenvironment. Specifically, we analyzed the percentages of CD4 T cells (Figure 6c), CD8 T cells (Figure 6d), TAM (CD45 + F4/80 + CD206+) (Figure 6e), M‐MDSC (CD45 + CD11b + Ly6C+) (Figure 6f), and PMN‐MDSC (CD45 + CD11b + Ly6G+) (Figure 6g). Notably, upon TAM and MDSC depletion, we observed a significant increase in the percentages of CD4 T cells and CD8 T cells, accompanied by a significant decrease in the percentages of TAM, M‐MDSC, and PMN‐MDSC (Figure 6c–g).

These findings suggested PRPS2 promoted the infiltration of TAM and MDSC into tumors. The use of clodronate liposomes and anti‐Gr‐1 antibody effectively reversed the phenotypic effects of PRPS2 overexpression, emphasizing the importance of TAM and MDSC in mediating the functions of PRPS2 in the tumor microenvironment.

## DISCUSSION

Our study investigated the role of PRPS2 in regulating the chemotaxis of TAM and MDSC within the tumor microenvironment. TAM and MDSC are known to contribute to the immunosuppressive milieu in cancer.^5^, ^7^, ^18^ Previous research has reported an association between increased TAM frequency and poor prognosis in several human tumors.^7^ MDSC, on the other hand, exhibit myeloid lineage characteristics and diverse cellular composition, enabling them to negatively regulate immune responses against cancer.^18^ While TAM and MDSC are traditionally considered distinct cell populations, their boundaries are not clearly defined, and they share common feature. Herein, we modulated the expression of PRPS2 in tumor cells and observed a significant impact on macrophage migration and MDSC number. These findings suggest that PRPS2 plays a regulatory role in modulating the chemotaxis of TAM and MDSC within the tumor microenvironment.

Prior investigations have established the essential role of the CCR2/CCL2 axis in the accumulation and functional specialization of MDSC and TAM.^19^ Within tumors, TAM are largely derived from CCR2+ monocytes, which are recruited from the bone marrow by CCL2.^8^ In another study, MDSC subsets had shown a positive correlation with CCL2.^20^ Herein, to elucidate the underlying mechanism, we explored the relationship between PRPS2 and the chemokine CCL2. Our results demonstrated a positive correlation between PRPS2 and CCL2, with PRPS2 knockout leading to decreased CCL2 levels, while PRPS2 overexpression resulted in increased CCL2 levels. Furthermore, the presence of a CCL2 antibody led to a reduction in the number of macrophages and MDSC, indicating the involvement of PRPS2‐mediated regulation of CCL2 in the chemotaxis of TAM and MDSC.

In order to validate our in vitro findings, we conducted an in vivo animal study to further investigate the role of PRPS2 in tumor development. Consistent with our in vitro observations, mice implanted with PRPS2 knockdown cells exhibited a significant reduction in tumor volume and weight, concomitant with a decrease in CCL2 levels within the tumor tissues. To understand the immune cell landscape in the tumor microenvironment, we assessed the composition of immune cells involved in both innate and adaptive immunity. Tumor tissues consist of various immune cell types, including T cells, TAM, and two distinct subsets of MDSC: M‐MDSC and PMN‐MDSC.^5^, ^7^, ^9^, ^18^ M‐MDSC, characterized by their monocytic lineage origin and identified as CD11b + Ly6C + Ly6G‐ cells with a monocyte‐like morphology, play a significant role in immunosuppression and regulation of immune responses within tumor tissues.^9^, ^18^ PMN‐MDSC, distinguished by their polymorphonuclear morphology and expression of markers such as CD11b + Ly6C‐Ly6G+, exhibit unique immunosuppressive functions similar to neutrophils and contribute to the inhibition of T cell responses and facilitation of immune evasion by tumors.^18^ TAM represents a significant population of immune cells within the tumor microenvironment, alongside MDSC. TAMs are often classified into two distinct populations: M1 macrophages, which exhibit proinflammatory properties, and M2 macrophages, which are associated with anti‐inflammatory functions.^5^, ^7^, ^9^, ^18^ While TAM and MDSC are recognized as distinct entities, the boundaries between them are not always clear, as they share many characteristics.^5^, ^7^, ^9^, ^18^ To comprehensively investigate the impact of PRPS2 on the recruitment of these immune cell populations, we employed fluorescence‐activated cell sorting analysis. Our results revealed that mice implanted with PRPS2 knockdown cells displayed an increased percentage of CD4+ and CD8+ T cells, accompanied by a decreased percentage of TAM, M‐MDSC, and PMN‐MDSC within the tumor tissues. In contrast, mice implanted with PRPS2‐overexpressing cells exhibited an opposite pattern in the composition of immune cells.

In addition to investigating the immune cell populations, we assessed the impact of PRPS2 on the regulation of specific biomarkers. S100A8 and S100A9 are recognized biomarkers associated with myeloid cells, including neutrophils and macrophages. Nos2 exhibits predominant expression in macrophages, dendritic cells, and neutrophils, while Arg1 is closely associated with macrophages and MDSC. Notably, our study revealed significant reductions in the mRNA levels of these biomarkers in the tumor tissues of mice implanted with LLC‐shPRPS2 cells. Conversely, there were substantial increases in the mRNA levels of these biomarkers in the tumor tissues of mice implanted with LLC‐PRPS2 cells. These findings collectively provide compelling evidence suggesting the potential role of PRPS2 in the regulation of immune cell infiltration within tumor microenvironments.

Furthermore, we investigated the impact of depleting TAM and MDSC on the phenotype resulting from PRPS2 overexpression. TAM and MDSC depletion resulted in reduced tumor volume and weight, along with increased percentages of T cells and decreased percentages of TAM, M‐MDSC, and PMN‐MDSC within the tumor microenvironment. These results indicate that TAMs and MDSCMDSC play a crucial role in mediating the functions of PRPS2 in the tumor microenvironment.

Finally, we have shown a positive correlation between PRPS2 and CCL2, and our in vivo findings further support the importance of PRPS2 in modulating immune cell infiltration into tumors. The use of clodronate liposomes and anti‐Gr‐1 antibody effectively reversed the phenotypic effects of PRPS2 overexpression, highlighting the significance of TAM and MDSC in mediating the functions of PRPS2 in the tumor microenvironment. While these findings are compelling, a limitation of this study was in the utilization of a single cell line (LLC cells) and its corresponding animal model. Thus, future investigations should validate these findings across various cell lines and animal models. Nonetheless, these findings enhance our comprehension of the regulatory mechanisms involved in immune cell recruitment in cancer and propose PRPS2 as a promising target for therapeutic interventions.

In conclusion, PRPS2 regulated the chemotaxis of TAM and MDSC in tumor cells by controlling CCL2 expression. In vivo experiments confirmed the involvement of PRPS2 in mediating CCL2 expression and regulating immune cell infiltration into tumors. Knockdown of CCL2 reversed the effects of PRPS2 overexpression. These findings underscored the crucial role of PRPS2 in reprogramming CCL2‐mediated TAM and MDSC functions, shaping the antitumor immune response in lung cancer.

## AUTHOR CONTRIBUTIONS

Qing Liu, Ningzi Wu and Peifeng Hou: Conducted the experiments. Qing Liu and Peifeng Hou: Analyzed the data. Peifeng Hou Supervised the study. Qing Liu, Ningzi Wu and Peifeng Hou: Wrote the manuscript.

## FUNDING INFORMATION

This study was supported by the Startup Fund for Scientific Research, Fujian Medical University (grant no. 2020QH1089), and Joint Funds for the Innovation of Science and Technology, Fujian Province (grant no. 2020Y9054).

## CONFLICT OF INTEREST STATEMENT

The author(s) declared no potential conflicts of interest with respect to the research, authorship, and/or publication of this article.

## References

[1] Minna JD , Roth JA , Gazdar AF . Focus on lung cancer. Cancer Cell. 2002;1:49–52. 12086887 10.1016/s1535-6108(02)00027-2

[2] Thandra KC , Barsouk A , Saginala K , Aluru JS , Barsouk A . Epidemiology of lung cancer. Contemporary Oncol/Współczesna Onkol. 2021;25:45–52. 10.5114/wo.2021.103829 PMC8063897 33911981

[3] Lu T , Yang X , Huang Y , Zhao M , Li M , Ma K , et al. Trends in the incidence, treatment, and survival of patients with lung cancer in the last four decades. Cancer Manag Res. 2019;11:943–953. 30718965 10.2147/CMAR.S187317 PMC6345192

[4] Gajewski TF , Schreiber H , Fu Y‐X . Innate and adaptive immune cells in the tumor microenvironment. Nat Immunol. 2013;14:1014–1022. 24048123 10.1038/ni.2703 PMC4118725

[5] Whiteside TL . The role of immune cells in the tumor microenvironment. The link between inflammation and cancer: wounds that do not heal. New York, NY: Springer; 2006. p. 103–124. 10.1007/0-387-26283-0_5 16610705

[6] Yan Y , Zhang L , Zuo Y , Qian H , Liu C . Immune checkpoint blockade in cancer immunotherapy: mechanisms, clinical outcomes, and safety profiles of PD‐1/PD‐L1 inhibitors. Arch Immunol Ther Exp (Warsz). 2020;68:1–15. 33185750 10.1007/s00005-020-00601-6

[7] Noy R , Pollard JW . Tumor‐associated macrophages: from mechanisms to therapy. Immunity. 2014;41:49–61. 25035953 10.1016/j.immuni.2014.06.010 PMC4137410

[8] Yang L , Zhang Y . Tumor‐associated macrophages: from basic research to clinical application. J Hematol Oncol. 2017;10:1–12. 28241846 10.1186/s13045-017-0430-2 PMC5329931

[9] Welch DR , Schissel DJ , Howrey RP , Aeed PA . Tumor‐elicited polymorphonuclear cells, in contrast to" normal" circulating polymorphonuclear cells, stimulate invasive and metastatic potentials of rat mammary adenocarcinoma cells. Proc Natl Acad Sci. 1989;86:5859–5863. 2762301 10.1073/pnas.86.15.5859 PMC297730

[10] Bakema JE , Ganzevles SH , Fluitsma DM , Schilham MW , Beelen RH , Valerius T , et al. Targeting FcαRI on polymorphonuclear cells induces tumor cell killing through autophagy. J. Immunol. 2011;187:726–732. 21653835 10.4049/jimmunol.1002581

[11] Cunningham JT , Moreno MV , Lodi A , Ronen SM , Ruggero D . Protein and nucleotide biosynthesis are coupled by a single rate‐limiting enzyme, PRPS2, to drive cancer. Cell. 2014;157:1088–1103. 24855946 10.1016/j.cell.2014.03.052 PMC4140650

[12] Liu G , Luo Y , Hou P . PRPS2 enhances resistance to cisplatin via facilitating exosomes‐mediated macrophage M2 polarization in non‐small cell lung cancer. Immunol Invest. 2022;51:1423–1436. 34251965 10.1080/08820139.2021.1952217

[13] Xu M , Wang Y , Xia R , Wei Y , Wei X . Role of the CCL2‐CCR2 signalling axis in cancer: mechanisms and therapeutic targeting. Cell Prolif. 2021;54:e13115. 34464477 10.1111/cpr.13115 PMC8488570

[14] Wang Y , Zhang X , Yang L , Xue J , Hu G . Blockade of CCL2 enhances immunotherapeutic effect of anti‐PD1 in lung cancer. J. Bone Oncol. 2018;11:27–32. 29892522 10.1016/j.jbo.2018.01.002 PMC5993943

[15] Xie M , Lin Z , Ji X , Luo X , Zhang Z , Sun M , et al. FGF19/FGFR4‐mediated elevation of ETV4 facilitates hepatocellular carcinoma metastasis by upregulating PD‐L1 and CCL2. J. Hepatol. 2023;79:109–125. 10.1016/j.jhep.2023.02.036 36907560

[16] Yang X , Lin Y , Shi Y , Li B , Liu W , Yin W , et al. FAP promotes immunosuppression by cancer‐associated fibroblasts in the tumor microenvironment via STAT3–CCL2 SignalingFAP via STAT3–CCL2 promote tumor immunosuppression. Cancer Res. 2016;76:4124–4135. 27216177 10.1158/0008-5472.CAN-15-2973

[17] Huang S , Wang Z , Zhou J , Huang J , Zhou L , Luo J , et al. EZH2 inhibitor GSK126 suppresses antitumor immunity by driving production of myeloid‐derived suppressor CellsGSK126 dampens antitumor immunity. Cancer Res. 2019;79:2009–2020. 30737232 10.1158/0008-5472.CAN-18-2395

[18] Kumar V , Patel S , Tcyganov E , Gabrilovich DI . The nature of myeloid‐derived suppressor cells in the tumor microenvironment. Trends Immunol. 2016;37:208–220. 26858199 10.1016/j.it.2016.01.004 PMC4775398

[19] Fei L , Ren X , Yu H , Zhan Y . Targeting the CCL2/CCR2 axis in cancer immunotherapy: one stone, three birds? Front Immunol. 2021;12:4657. 10.3389/fimmu.2021.771210 PMC8596464 34804061

[20] Chun E , Lavoie S , Michaud M , Gallini CA , Kim J , Soucy G , et al. CCL2 promotes colorectal carcinogenesis by enhancing polymorphonuclear myeloid‐derived suppressor cell population and function. Cell Rep. 2015;12:244–257. 26146082 10.1016/j.celrep.2015.06.024 PMC4620029

